# Crystal structure of (4-fluoro­phenyl-κ*C* ^1^)iodido­(*N*,*N*,*N*′,*N*′-tetra­methyl­ethylenedi­amine-κ^2^ *N*,*N*′)palladium(II)

**DOI:** 10.1107/S2056989015008014

**Published:** 2015-04-30

**Authors:** Jin-Jin Yan, Chang-Ge Zheng

**Affiliations:** a School of Chemical and Material Engineering, Jiangnan University, 1800 Lihu Road, Wuxi, Jiangsu Province 214122, People’s Republic of China

**Keywords:** crystal structure, palladium(II) complex, tetra­methyl­ethylenedi­amine, square-planar coordination, single-crystal X-ray study, hydrogen bonding

## Abstract

In the title compound, [Pd(C_6_H_4_F)I(C_6_H_16_N_2_)], the Pd^II^ atom is coordinated by two N atoms from the *N*,*N*,*N*′,*N*′-tetra­methyl­ethylenedi­amine ligand, a C atom of the 4-fluoro­phenyl group and an iodide ligand in a distorted square-planar geometry, with an average deviation from the least-squares plane through the ligand donor atoms of 0.0159 (2) Å. The angles about the Pd^II^ atom range from 83.35 (16) to 178.59 (11)°. In the crystal, weak C—H⋯F and C—H⋯I hydrogen bonds link the mol­ecules into sheets in the *bc* plane.

## Related literature   

For related palladium complexes with Pd^II^—I bonds, see: Racowski *et al.* (2011 ▸); Grushin & Marshall (2006 ▸); Ball *et al.* (2010 ▸). For the role of iodido palladium aryl complexes in coupling reactions, see: Hartwig (2008 ▸); Wu *et al.* (2010 ▸); and as precursors to tri­fluoro­methyl palladium aryl complexes, see: Maleckis & Sanford (2011 ▸); Ball *et al.* (2010 ▸); Ye *et al.* (2010 ▸); Racowski *et al.* (2011 ▸); Ball *et al.* (2011 ▸); Grushin & Marshall (2006 ▸); Du & Zheng (2014 ▸). For a related palladium complex with a Pd^II^—C bond, see: Du & Zheng (2014 ▸).
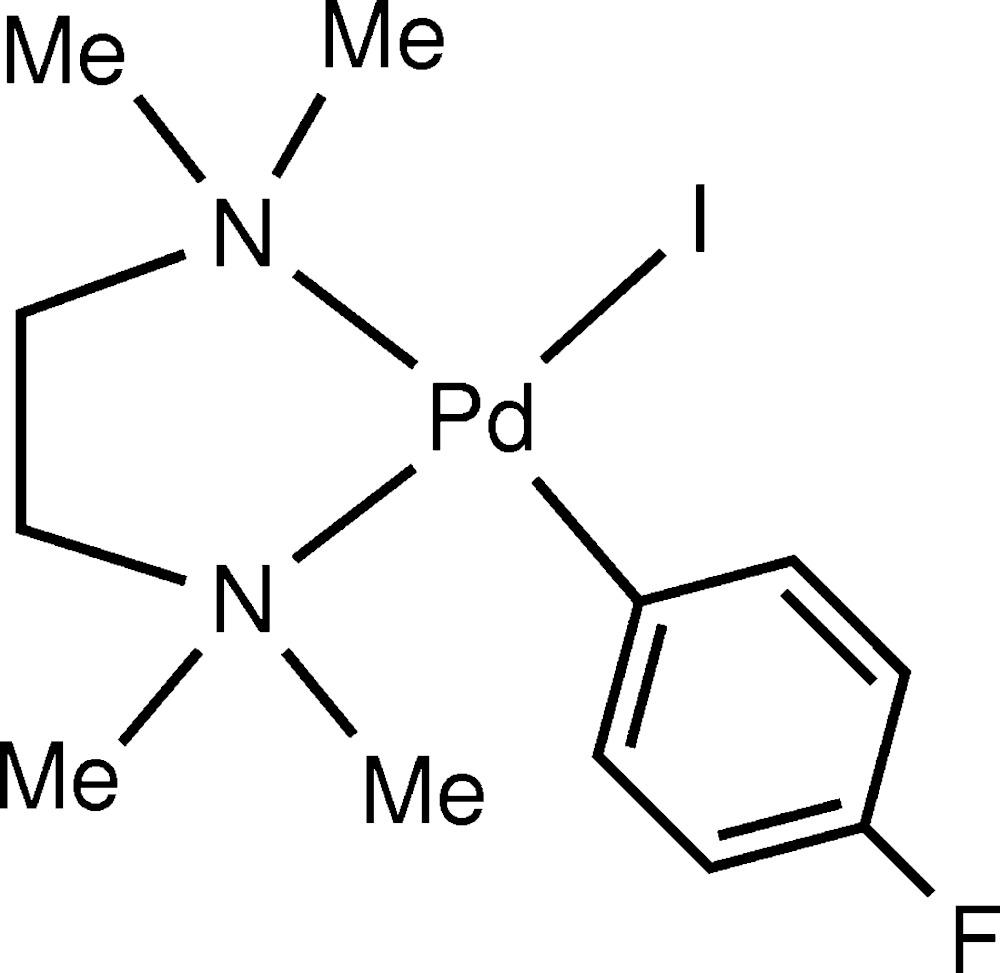


## Experimental   

### Crystal data  

[Pd(C_6_H_4_F)I(C_6_H_16_N_2_)]
*M* *_r_* = 444.60
Monoclinic, 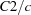

*a* = 9.456 (2) Å
*b* = 12.802 (3) Å
*c* = 24.953 (5) Å
β = 93.152 (2)°
*V* = 3015.9 (11) Å^3^
*Z* = 8
Mo *K*α radiation
μ = 3.27 mm^−1^
*T* = 296 K
0.26 × 0.24 × 0.20 mm

### Data collection  

Bruker APEXII CCD diffractometer
Absorption correction: multi-scan (*SADABS*; Bruker, 2007 ▸) *T*_min_ = 0.483, *T*_max_ = 0.561
10757 measured reflections
2827 independent reflections
2736 reflections with *I* > 2σ(*I*)
*R*_int_ = 0.058

### Refinement  

*R*[*F* ^2^ > 2σ(*F* ^2^)] = 0.039
*wR*(*F* ^2^) = 0.120
*S* = 1.00
2827 reflections
158 parameters
H-atom parameters constrained
Δρ_max_ = 1.15 e Å^−3^
Δρ_min_ = −1.76 e Å^−3^

### 

Data collection: *APEX2* (Bruker, 2007 ▸); cell refinement: *SAINT* (Bruker, 2007 ▸); data reduction: *SAINT*; program(s) used to solve structure: *SHELXS97* (Sheldrick, 2008 ▸); program(s) used to refine structure: *SHELXL97* (Sheldrick, 2008 ▸); molecular graphics: *SHELXTL* (Sheldrick, 2008 ▸); software used to prepare material for publication: *SHELXTL*.

## Supplementary Material

Crystal structure: contains datablock(s) I, New_Global_Publ_Block. DOI: 10.1107/S2056989015008014/sj5451sup1.cif

**Fig. 1.**
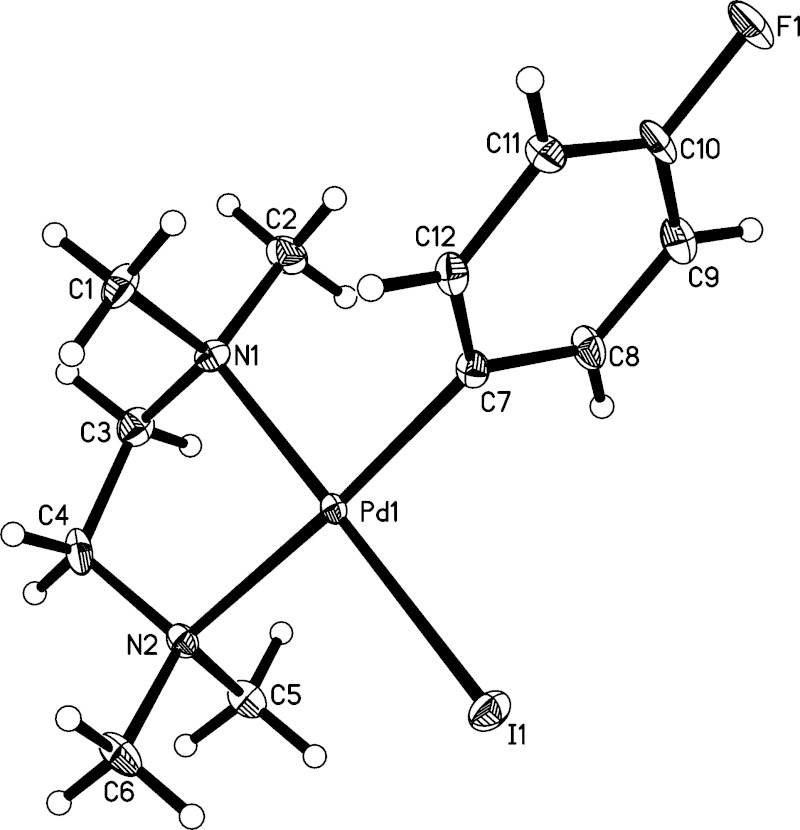
The molecular structure of [(tmeda)Pd(p-FPh)(I)], with the atom-numbering scheme and 30% probability displacement ellipsoids.

**Fig. 2.**
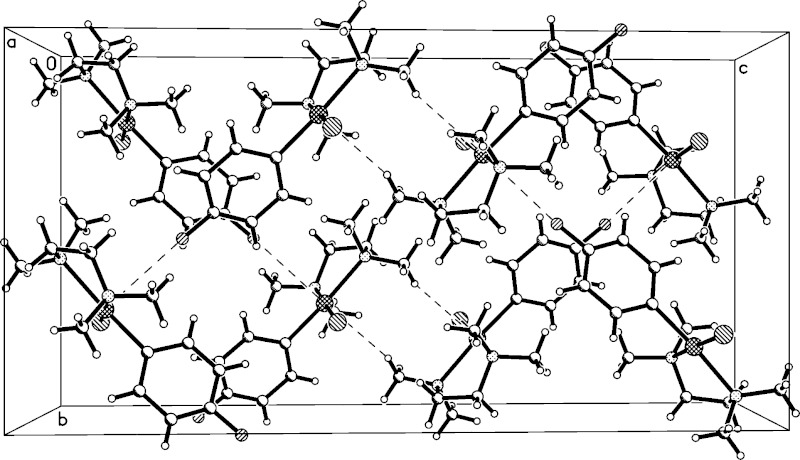
The molecular packing of [(tmeda)Pd(p-FPh)(I)] viewed along the a axis showing C—H···F and C—H···I interactions as dashed lines.

Structure factors: contains datablock(s) I. DOI: 10.1107/S2056989015008014/sj5451Isup2.hkl

p . DOI: 10.1107/S2056989015008014/sj5451fig1.tif
The mol­ecular structure of [(tmeda)Pd(*p*-FPh)(I)], with the atom-numbering scheme and 30% probability displacement ellipsoids.

p a . DOI: 10.1107/S2056989015008014/sj5451fig2.tif
The mol­ecular packing of [(tmeda)Pd(*p*-FPh)(I)] viewed along the *a* axis showing C—H⋯F and C—H⋯I inter­actions as dashed lines.

CCDC reference: 1061123

Additional supporting information:  crystallographic information; 3D view; checkCIF report

## Figures and Tables

**Table 1:** Selected bond lengths ()

Pd1C7	1.990(5)
Pd1N1	2.138(4)
Pd1N2	2.198(4)
Pd1I1	2.5823(7)

**Table 2:** Hydrogen-bond geometry (, )

*D*H*A*	*D*H	H*A*	*D* *A*	*D*H*A*
C2H2*A*F1^i^	0.96	2.57	3.445(6)	151
C5H5*C*F1^i^	0.96	2.59	3.412(5)	144
C1H1*C*I1^ii^	0.96	3.19	4.050(5)	150
C4H4*B*I1^iii^	0.97	3.24	4.017(5)	138

Symmetry codes: (i) 
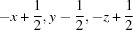
; (ii) 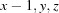; (iii) 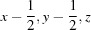.

## supplementary crystallographic information

### S1. Comment

Halogen metal complexes, especially iodido palladium aryl complexes, have attracted much attention because of their important roles in coupling reactions (Hartwig, 2008; Wu *et al.*,2010). They are also significant precursors of tri­fluoro­methyl palladium aryl complexes, which are used in C—H tri­fluoro­methyl­ation reactions (Maleckis & Sanford,2011; Ball *et al.*,2010; Ye *et al.*,2010; Racowski *et al.*,2011; Ball *et al.*,2011;Grushin & Marshall, 2006;Du & Zheng, 2014).

Single-crystal X-ray diffraction of the title compound reveals that the Pd^II^ centre in [(tmeda)Pd(*p*-FPh)I] is four-coordinate. As shown in Fig. 1, the asymmetric unit comprises a Pd^II^ cation, a tmeda ligand binding through N1 and N2, a *p*-FC_6_H_4_ group binding through C12 and the iodide anion, I1. For selected bond lengths, see Table 2. The Pd–I1 bond length is 2.5823 (7) Å, which is shorter than that for the complex [(dppe)Pd(CF_3_)I] (dppe = 1,2-bis­(di­phenyl­phosphino)ethane) ( Grushin & Marshall, 2006). The Pd–C bond length (1.990 (5) Å) is compares well to that in the related complex [[(tmeda)Pd(*p*-FPh)(CF_3_)] (2.004 (3) Å) (Du & Zheng, 2014). Fig. 2 shows the molecular packing of the title compound, viewed along the *a* axis. In the crystal, weak C—H···F and C—H···I hydrogen bonds link the molecules into sheets in the *bc* plane (Table 1).

### S2. Synthesis and crystallization

Under nitro­gen, Pd(dba)_2_ (915.72 mg, 1 mmol, 1 equiv) was placed into a 250 mL round bottom flask and dissolved in THF (30 mL). TMEDA (631.35 mg, 5.2 mmol, 5.2 equiv) was added, and the resulting mixture was stirred at 25 °C for 15 min. 4-Fluoro­iodo­benzene (950 mg, 4 mmol, 4 equiv) was added, and the reaction was heated at 60 °C for 30 min. The reaction mixture was filtered in air through a plug of Celite, and the solvent was removed under reduced pressure. The resulting solid was washed with hexane (3 × 30 mL) and di­ethyl ether (3 × 50 mL) to remove all residual di­benzyl­idene acetone (dba). The product was then dried in vacuo. Yield: 560 mg (65%) of an orange solid. 40 mg of [(tmeda)Pd(*p*-FPh)(I)] were put into a 10 ml transparent bottle and dissolved in CH_2_Cl_2_ (2 mL). The neck of the bottle was sealed with plastic wrap, and the bottle was put inside a wide mouth transparent bottle containing 15 mL di­ethyl ether. Orange acicular single crystals of [(tmeda)Pd(*p*-FPh)(I)] were obtained after 3 days.

### S3. Refinement

The H atoms bound to C were introduced at calculated positions and refined using a riding model, with U_iso_(H) = 1.2U_eq_–1.5U_eq_(C) with C–H distances of 0.93–0.97 Å.

### Crystal data

**Table:**

[Pd(C_6_H_4_F)I(C_6_H_16_N_2_)]	*F*(000) = 1712
*M**_r_* = 444.60	*D*_x_ = 1.958 Mg m^−^^3^
Monoclinic, *C*2/*c*	Mo *K*α radiation, λ = 0.71073 Å
Hall symbol: -C 2yc	Cell parameters from 8524 reflections
*a* = 9.456 (2) Å	θ = 2.7–28.3°
*b* = 12.802 (3) Å	µ = 3.27 mm^−^^1^
*c* = 24.953 (5) Å	*T* = 296 K
β = 93.152 (2)°	Block, colorless
*V* = 3015.9 (11) Å^3^	0.26 × 0.24 × 0.20 mm
*Z* = 8	

### Data collection

**Table:**

Bruker APEXII CCD diffractometer	2827 independent reflections
Radiation source: fine-focus sealed tube	2736 reflections with *I* > 2σ(*I*)
Graphite monochromator	*R*_int_ = 0.058
φ and ω scans	θ_max_ = 25.6°, θ_min_ = 2.7°
Absorption correction: multi-scan (*SADABS*; Bruker, 2007)	*h* = −11→11
*T*_min_ = 0.483, *T*_max_ = 0.561	*k* = −15→11
10757 measured reflections	*l* = −30→30

### Refinement

**Table:**

Refinement on *F*^2^	Primary atom site location: structure-invariant direct methods
Least-squares matrix: full	Secondary atom site location: difference Fourier map
*R*[*F*^2^ > 2σ(*F*^2^)] = 0.039	Hydrogen site location: inferred from neighbouring sites
*wR*(*F*^2^) = 0.120	H-atom parameters constrained
*S* = 1.00	*w* = 1/[σ^2^(*F*_o_^2^) + (0.0848*P*)^2^ + 14.1377*P*] where *P* = (*F*_o_^2^ + 2*F*_c_^2^)/3
2827 reflections	(Δ/σ)_max_ = 0.001
158 parameters	Δρ_max_ = 1.15 e Å^−^^3^
0 restraints	Δρ_min_ = −1.76 e Å^−^^3^

### Special details

**Table:**

Geometry. All e.s.d.'s (except the e.s.d. in the dihedral angle between two l.s. planes) are estimated using the full covariance matrix. The cell e.s.d.'s are taken into account individually in the estimation of e.s.d.'s in distances, angles and torsion angles; correlations between e.s.d.'s in cell parameters are only used when they are defined by crystal symmetry. An approximate (isotropic) treatment of cell e.s.d.'s is used for estimating e.s.d.'s involving l.s. planes.
Refinement. Refinement of *F*^2^ against ALL reflections. The weighted *R*-factor *wR* and goodness of fit *S* are based on *F*^2^, conventional *R*-factors *R* are based on *F*, with *F* set to zero for negative *F*^2^. The threshold expression of *F*^2^ > σ(*F*^2^) is used only for calculating *R*-factors(gt) *etc*. and is not relevant to the choice of reflections for refinement. *R*-factors based on *F*^2^ are statistically about twice as large as those based on *F*, and *R*- factors based on ALL data will be even larger.

### Fractional atomic coordinates and isotropic or equivalent isotropic displacement parameters (Å^2^)

**Table:**

	*x*	*y*	*z*	*U*_iso_*/*U*_eq_	
Pd1	0.26525 (4)	0.69994 (3)	0.390741 (13)	0.01328 (16)	
I1	0.52911 (4)	0.74248 (3)	0.412548 (15)	0.02671 (17)	
N2	0.2556 (4)	0.5701 (3)	0.44807 (16)	0.0182 (8)	
N1	0.0479 (4)	0.6607 (3)	0.37289 (17)	0.0182 (9)	
C4	0.1050 (6)	0.5433 (4)	0.4489 (2)	0.0273 (12)	
H4A	0.0597	0.5893	0.4737	0.033*	
H4B	0.0956	0.4722	0.4617	0.033*	
C7	0.2585 (5)	0.8106 (4)	0.3346 (2)	0.0188 (10)	
C9	0.2906 (6)	0.8594 (5)	0.2421 (2)	0.0304 (12)	
H9	0.3254	0.8453	0.2087	0.036*	
C5	0.3356 (6)	0.4817 (4)	0.4266 (2)	0.0243 (11)	
H5A	0.3259	0.4216	0.4491	0.036*	
H5B	0.4339	0.5002	0.4258	0.036*	
H5C	0.2990	0.4659	0.3908	0.036*	
C12	0.1910 (5)	0.9053 (4)	0.3415 (2)	0.0206 (10)	
H12	0.1600	0.9221	0.3751	0.025*	
C11	0.1680 (6)	0.9762 (4)	0.2996 (2)	0.0245 (11)	
H11	0.1202	1.0386	0.3047	0.029*	
C8	0.3101 (6)	0.7898 (4)	0.2838 (2)	0.0260 (12)	
H8	0.3584	0.7277	0.2783	0.031*	
C1	−0.0461 (6)	0.7340 (5)	0.3997 (3)	0.0280 (12)	
H1A	−0.0455	0.8004	0.3819	0.042*	
H1B	−0.0130	0.7425	0.4365	0.042*	
H1C	−0.1407	0.7066	0.3982	0.042*	
C2	0.0035 (6)	0.6583 (5)	0.3150 (2)	0.0296 (12)	
H2A	0.0678	0.6153	0.2963	0.044*	
H2B	0.0045	0.7280	0.3008	0.044*	
H2C	−0.0905	0.6301	0.3105	0.044*	
C10	0.2174 (6)	0.9516 (4)	0.2509 (2)	0.0256 (11)	
C6	0.3130 (7)	0.5902 (5)	0.5036 (2)	0.0298 (12)	
H6A	0.2608	0.6459	0.5190	0.045*	
H6B	0.4110	0.6094	0.5030	0.045*	
H6C	0.3043	0.5281	0.5248	0.045*	
C3	0.0317 (6)	0.5531 (4)	0.3942 (2)	0.0258 (11)	
H3A	0.0719	0.5033	0.3700	0.031*	
H3B	−0.0681	0.5371	0.3964	0.031*	
F1	0.1947 (4)	1.0191 (3)	0.20905 (13)	0.0387 (9)	

### Atomic displacement parameters (Å^2^)

**Table:**

	*U*^11^	*U*^22^	*U*^33^	*U*^12^	*U*^13^	*U*^23^
Pd1	0.0155 (2)	0.0132 (2)	0.0113 (2)	0.00004 (12)	0.00249 (15)	0.00002 (12)
I1	0.0180 (2)	0.0254 (2)	0.0364 (3)	−0.00383 (12)	−0.00055 (17)	0.00083 (14)
N2	0.024 (2)	0.017 (2)	0.014 (2)	0.0018 (17)	0.0023 (16)	−0.0014 (16)
N1	0.017 (2)	0.015 (2)	0.022 (2)	0.0013 (16)	0.0000 (16)	−0.0027 (17)
C4	0.036 (3)	0.024 (3)	0.024 (3)	0.002 (2)	0.016 (2)	0.004 (2)
C7	0.021 (2)	0.022 (2)	0.014 (2)	−0.002 (2)	0.0019 (19)	−0.0040 (19)
C9	0.038 (3)	0.034 (3)	0.020 (3)	−0.004 (3)	0.007 (2)	0.003 (2)
C5	0.035 (3)	0.018 (2)	0.020 (3)	0.001 (2)	0.000 (2)	−0.001 (2)
C12	0.024 (2)	0.021 (3)	0.017 (2)	−0.006 (2)	0.0037 (19)	−0.002 (2)
C11	0.027 (3)	0.023 (3)	0.024 (3)	0.001 (2)	0.000 (2)	0.006 (2)
C8	0.035 (3)	0.025 (3)	0.019 (3)	0.003 (2)	0.010 (2)	0.001 (2)
C1	0.021 (3)	0.026 (3)	0.038 (3)	0.004 (2)	0.011 (2)	−0.003 (2)
C2	0.034 (3)	0.037 (3)	0.018 (3)	−0.003 (3)	−0.005 (2)	0.000 (2)
C10	0.037 (3)	0.025 (3)	0.015 (2)	−0.009 (2)	−0.001 (2)	0.009 (2)
C6	0.047 (3)	0.028 (3)	0.014 (3)	0.004 (3)	0.001 (2)	0.000 (2)
C3	0.024 (3)	0.021 (3)	0.032 (3)	−0.004 (2)	0.003 (2)	0.000 (2)
F1	0.053 (2)	0.039 (2)	0.0244 (18)	−0.0003 (17)	0.0019 (15)	0.0191 (15)

### Geometric parameters (Å, º)

**Table:**

Pd1—C7	1.990 (5)	C5—H5B	0.9600
Pd1—N1	2.138 (4)	C5—H5C	0.9600
Pd1—N2	2.198 (4)	C12—C11	1.393 (7)
Pd1—I1	2.5823 (7)	C12—H12	0.9300
N2—C4	1.466 (7)	C11—C10	1.361 (8)
N2—C5	1.479 (6)	C11—H11	0.9300
N2—C6	1.482 (7)	C8—H8	0.9300
N1—C1	1.477 (7)	C1—H1A	0.9600
N1—C2	1.482 (7)	C1—H1B	0.9600
N1—C3	1.488 (7)	C1—H1C	0.9600
C4—C3	1.501 (8)	C2—H2A	0.9600
C4—H4A	0.9700	C2—H2B	0.9600
C4—H4B	0.9700	C2—H2C	0.9600
C7—C12	1.385 (8)	C10—F1	1.364 (6)
C7—C8	1.410 (7)	C6—H6A	0.9600
C9—C8	1.376 (8)	C6—H6B	0.9600
C9—C10	1.392 (9)	C6—H6C	0.9600
C9—H9	0.9300	C3—H3A	0.9700
C5—H5A	0.9600	C3—H3B	0.9700
			
C7—Pd1—N1	91.56 (19)	C7—C12—C11	122.2 (5)
C7—Pd1—N2	174.34 (18)	C7—C12—H12	118.9
N1—Pd1—N2	83.35 (16)	C11—C12—H12	118.9
C7—Pd1—I1	89.51 (15)	C10—C11—C12	118.1 (5)
N1—Pd1—I1	178.59 (11)	C10—C11—H11	120.9
N2—Pd1—I1	95.55 (11)	C12—C11—H11	120.9
C4—N2—C5	110.1 (4)	C9—C8—C7	121.4 (5)
C4—N2—C6	109.4 (4)	C9—C8—H8	119.3
C5—N2—C6	107.6 (4)	C7—C8—H8	119.3
C4—N2—Pd1	105.2 (3)	N1—C1—H1A	109.5
C5—N2—Pd1	107.6 (3)	N1—C1—H1B	109.5
C6—N2—Pd1	116.8 (3)	H1A—C1—H1B	109.5
C1—N1—C2	108.0 (4)	N1—C1—H1C	109.5
C1—N1—C3	110.4 (4)	H1A—C1—H1C	109.5
C2—N1—C3	107.5 (4)	H1B—C1—H1C	109.5
C1—N1—Pd1	110.6 (3)	N1—C2—H2A	109.5
C2—N1—Pd1	115.2 (3)	N1—C2—H2B	109.5
C3—N1—Pd1	105.0 (3)	H2A—C2—H2B	109.5
N2—C4—C3	111.5 (4)	N1—C2—H2C	109.5
N2—C4—H4A	109.3	H2A—C2—H2C	109.5
C3—C4—H4A	109.3	H2B—C2—H2C	109.5
N2—C4—H4B	109.3	C11—C10—F1	119.3 (5)
C3—C4—H4B	109.3	C11—C10—C9	122.4 (5)
H4A—C4—H4B	108.0	F1—C10—C9	118.3 (5)
C12—C7—C8	117.4 (5)	N2—C6—H6A	109.5
C12—C7—Pd1	122.3 (4)	N2—C6—H6B	109.5
C8—C7—Pd1	120.0 (4)	H6A—C6—H6B	109.5
C8—C9—C10	118.4 (5)	N2—C6—H6C	109.5
C8—C9—H9	120.8	H6A—C6—H6C	109.5
C10—C9—H9	120.8	H6B—C6—H6C	109.5
N2—C5—H5A	109.5	N1—C3—C4	110.6 (5)
N2—C5—H5B	109.5	N1—C3—H3A	109.5
H5A—C5—H5B	109.5	C4—C3—H3A	109.5
N2—C5—H5C	109.5	N1—C3—H3B	109.5
H5A—C5—H5C	109.5	C4—C3—H3B	109.5
H5B—C5—H5C	109.5	H3A—C3—H3B	108.1
			
C7—Pd1—N2—C4	−35.7 (19)	N1—Pd1—C7—C12	74.4 (4)
N1—Pd1—N2—C4	−9.8 (3)	N2—Pd1—C7—C12	100.1 (18)
I1—Pd1—N2—C4	171.0 (3)	I1—Pd1—C7—C12	−106.5 (4)
C7—Pd1—N2—C5	81.7 (18)	N1—Pd1—C7—C8	−98.7 (4)
N1—Pd1—N2—C5	107.5 (3)	N2—Pd1—C7—C8	−73.0 (19)
I1—Pd1—N2—C5	−71.6 (3)	I1—Pd1—C7—C8	80.4 (4)
C7—Pd1—N2—C6	−157.2 (17)	C8—C7—C12—C11	3.0 (8)
N1—Pd1—N2—C6	−131.4 (4)	Pd1—C7—C12—C11	−170.3 (4)
I1—Pd1—N2—C6	49.5 (4)	C7—C12—C11—C10	−1.7 (8)
C7—Pd1—N1—C1	−81.4 (4)	C10—C9—C8—C7	−0.3 (9)
N2—Pd1—N1—C1	101.0 (4)	C12—C7—C8—C9	−1.9 (8)
I1—Pd1—N1—C1	139 (4)	Pd1—C7—C8—C9	171.5 (5)
C7—Pd1—N1—C2	41.4 (4)	C12—C11—C10—F1	179.1 (5)
N2—Pd1—N1—C2	−136.1 (4)	C12—C11—C10—C9	−0.7 (8)
I1—Pd1—N1—C2	−98 (5)	C8—C9—C10—C11	1.7 (9)
C7—Pd1—N1—C3	159.5 (3)	C8—C9—C10—F1	−178.1 (5)
N2—Pd1—N1—C3	−18.1 (3)	C1—N1—C3—C4	−75.3 (6)
I1—Pd1—N1—C3	20 (5)	C2—N1—C3—C4	167.1 (4)
C5—N2—C4—C3	−78.6 (5)	Pd1—N1—C3—C4	43.9 (5)
C6—N2—C4—C3	163.2 (5)	N2—C4—C3—N1	−57.3 (6)
Pd1—N2—C4—C3	37.0 (5)		

### Hydrogen-bond geometry (Å, º)

**Table:**

*D*—H···*A*	*D*—H	H···*A*	*D*···*A*	*D*—H···*A*
C2—H2*A*···F1^i^	0.96	2.57	3.445 (6)	151
C5—H5*C*···F1^i^	0.96	2.59	3.412 (5)	144
C1—H1*C*···I1^ii^	0.96	3.19	4.050 (5)	150
C4—H4*B*···I1^iii^	0.97	3.24	4.017 (5)	138

Symmetry codes: (i) −*x*+1/2, *y*−1/2, −*z*+1/2; (ii) *x*−1, *y*, *z*; (iii) *x*−1/2, *y*−1/2, *z*.
